# The proline effect and the tryptophan immonium ion assist in de novo sequencing of adipokinetic hormones

**DOI:** 10.1038/s41598-023-38056-2

**Published:** 2023-07-05

**Authors:** Simone König, Heather G. Marco, Gerd Gäde

**Affiliations:** 1 IZKF Core Unit Proteomics, University of Münster, 48149 Münster, Germany; 2 Department of Biological Sciences, University of Cape Town, ZA-7701 Rondebosch, Cape Town, South Africa

**Keywords:** Analytical chemistry, Biochemistry, Chemical biology, Biochemistry, Biological techniques, Developmental biology, Evolution, Zoology

## Abstract

Adipokinetic hormones (AKHs) in Arthopoda are characterized by special sequence features including limited choices of amino acid residues in certain positions, such as Trp in position 8. Over 100 different AKHs have been described, but de novo sequencing of novel peptide hormones can be a challenge. In a project of analyzing corpora cardiaca extracts from two fly species, two different moths, a termite and a beetle for their AKHs, we noted specific patterns in the fragmentation spectra of octapeptides in electrospray Q-TOF experiments resulting from the presence of Pro in position 6. The preference for cleavage N-terminal to Pro residues created an abundant y_3_″-ion, which, in conjunction with the two b-ions resulting from the fragmentation before and after Pro, provided a marker pattern. As Pro6 occurs in about 61% of known AKHs, this information is highly relevant for sequence elucidation. Moreover, the default presence of Trp8 allowed the use of its immonium ion for AKH candidate identification. In addition, we assembled the known AKH sequences from the literature and sequences of AKH-type format found in the Uniprot database in a single online resource. These efforts assisted in the analysis workflow and led to the assignment of two novel AKHs and evidence for the presence of Melme-CC and Dorpa-AKH in the corpus cardiacum of the scarab beetle *Sinodendron cylindricum*.

## Introduction

Adipokinetic hormones (AKHs) are important regulators of metabolism in insects (for a recent review see^1^). One to five, depending on the species, of these peptides are synthesized in neurosecretory cells of the neurohemal organs, the corpora cardiaca (CC). The peptides are characterized by a chain length of 8–10 amino acid residues, an N-terminal pyroglutamate residue (abbreviated Pyr, pGlu, pE or pQ), an amidated C-terminus, and favored amino acids in some positions such as Leu, Ile, Val, Phe or Tyr in position 2; Asn or Thr in position 3, Phe or Tyr in position 4, Ser or Thr in position 5, often Pro in position 6 and Gly in position 7, and Trp in position 8. About one third of the known AKHs extend beyond eight amino acid residues and have mostly Gly in position 9. There are exceptions to these rules like Met in position 4 (Trifa-CC^2^) or Val in position 9 as in the AKH of the Asian long-horned beetle, *Anoplophora glabripennis*^3^. Additional posttranslational modifications have been described, such as mannosylated Trp in Carmo-HrTH-I^1^, phosphorylated Thr in Trifa-CC^2^ and Pro isomerisation in Placa-HrTH I/II^1^.

Traditionally, AKHs have been traced based on their aromatic amino acids and in particular the conventional Trp8 residue using UV and fluorescence detection^1^. In recent years, mass spectrometry (MS) in conjunction with liquid chromatography (LC) has increasingly been employed for AKH identification, which delivers confident mass and sequence information. However, more often than not, a selective Trp detector is not available in such an experimental setting, and it can then be difficult to locate the AKHs among all other signals.

**Figure 1:**
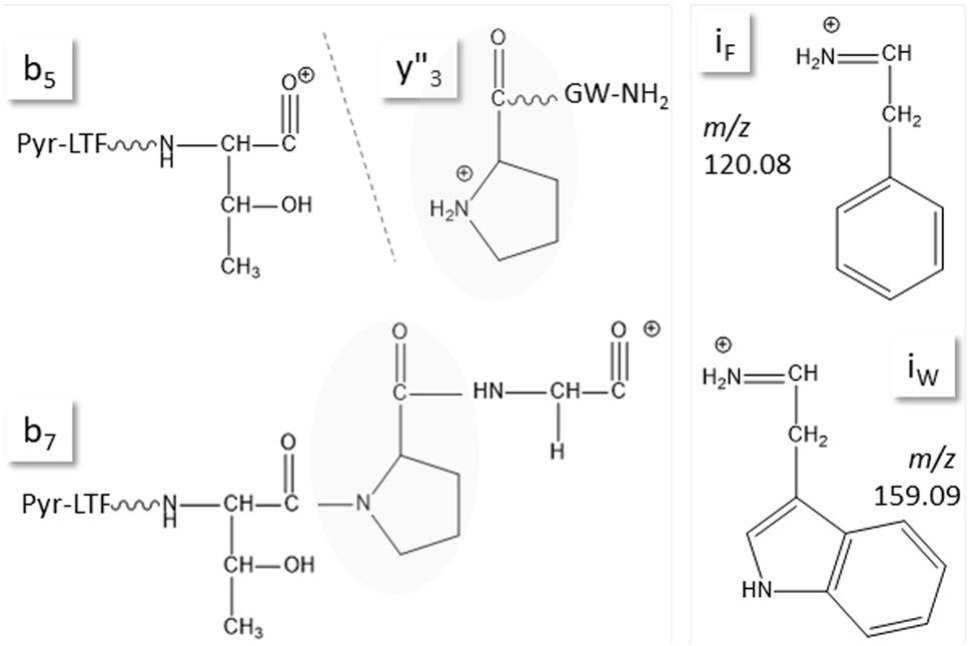
Structures of the y”_3_, b_5_, and b_7_ ions in 8-amino acid residue AKHs as well as the Trp (i_W_) and Phe (i_F_) immonium ions. For simple b ions, cyclic oxazolone structures have been proposed^6^ instead of the exemplary fragmentation product shown here. Ion assignment follows Waters instrument MassLynx software output as shown in Supplementary File, sheet “MSMS ions”.

In the course of analyzing AKHs from different insect species we had learned previously that AKHs may show characteristic ionization behavior, depending on the instrumentation, such as abundant sodiated and potassiated parent ions in MALDI and ion trap MS^4^. During the study of CC extracts from two dipteran species (the robber fly *Pegesimallus tapulus*, family Asilidae, and the horse fly *Haematopota pluvialis*, family Tabanidae), two different moths (the European maize borer *Ostrinia nubilalis* and the garden grass-veneer *Chrysoteuchia culmella*, both family Crambidae), and the termite *Kalotermes flavicollis*, family Kalotermitidae, (see ref.^5^), as well as the beetle *Sinodendron cylindricum* (family Lucanidae, this study), with reversed-phase (RP)LC coupled to high-resolution (HR)MS, we noticed distinctive features of the ion patterns associated to AKH fragmentation, which helped in de novo sequencing. An ion trio was characteristically observed during the fragmentation of AKHs that contained Pro in position 6 of the peptide chain; this can serve as a marker for sequence assignment of new AKHs based on peptide MS/MS data. The trio of ions arises from the special way in which an AKH with Pro6 breaks during collision-induced dissociation (CID) and is a consequence of the rigid ring structure of this amino acid residue. Trp and Phe immonium ions are other useful diagnostic markers during AKH screening in a crude CC extract. Immonium ions are small diagnostic ions observed at the low end of the fragment ion spectrum, which still contain the amino acid side chain and thus indicate the presence of a certain amino acid residue (Fig. 1).

Furthermore, the identification of AKHs can rely on prior knowledge of which amino acid compositions might be present. For this purpose, we had previously programmed the web tool MAPSP (Mass Analysis Peptide Sequence Prediction^7^), which calculates possible sequences based on measured mass and available amino acid information. However, the number of sequence combinations can still be too large to arrive at useful conclusions so that it is of advantage to compare postulated sequences with the peptide and protein knowledgebase, which is currently rather diverse and difficult to peruse. On the one hand, there are individual reports for discovered AKHs and reviews on selected hormone groups and, on the other hand, there are predicted AKHs by genomic and transcriptomic work stored in databases. The matter is further compounded by an irregular, inconsistent nomenclature applied to the identified sequences so that the same peptide sequence can have different names simply because it is found in multiple species. For example, one set of scientists calls the peptide pQLTFSPDW-NH_2_ “Phote-HrTH” irrespective in which insects it is sequenced and what function it has (i.e. using the name of the species in which the peptide was first fully structurally identified (*Phormia terranovae*) and the demonstrated peptide function (hypertrehalosemic hormone) in that species^8^. Other researchers may use a different name for this exact same peptide sequence when it occurs in another species, for example, Phote-HrTH was called “Drome-AKH”, when it was found in *Drosophila melanogaster* (see DINeR database^9^ also for other examples). In an effort to further support AKH de novo sequencing by MS, we aimed to assemble lists of both the validated AKHs and AKH-type sequences extracted from Uniprot and to unite them in a searchable collection as a single resource of sequence information. This archive can be interrogated during the generation of peptide sequence hypotheses in order to find likely candidates.

In the current work, we direct the attention of the interested researcher to the special MS ionization and fragmentation behavior of AKHs, which results from conserved amino acids such as Pro6 and Trp8. This knowledge assists in the assignment of newly detected AKHs in conjunction with the above-mentioned AKH database. We discuss the experimental workflow of CC extract analysis using HRMS and describe the identification of novel AKHs detected in the CC extracts of two fly species (*P. tapulus*, *H. pluvialis*). Moreover, we present evidence for the presence of Melme-CC and Dorpa-AKH in the CC of the scarab beetle *Sinodendron cylindricum.*

## Material and methods

*Insects and CC preparation:* The preparation of specimens of *K. flavicollis, H. pluvialis, P. tapulus, O. nubilalis,* and *C. culmella* was described before^5^. Adults of the rhinoceros stag beetle *S. cylindricum* (Coleoptera, Polyphaga, Scarabaeoidea, Lucanidae) were collected in a decaying wood stump in the vicinity of Jena (Thuringia, Germany). CC were dissected with the aid of a stereomicroscope at 20- to 40-fold magnification from adult insects of indeterminate age. The glands were placed into a microcentrifuge tube containing 80% v/v methanol, extracted by approved methods^1,5^, and dried in a vacuum centrifuge. The samples were re-dissolved in 10 µl methanol followed by 10 µl 0.1% formic acid containing 5% acetonitrile.

*LC–MS/MS*: Synapt G2 Si coupled to M-Class nano UPLC (Waters Corp., Manchester, UK) was employed using C18 µPAC columns (trapping and 50 cm analytical; PharmaFluidics, Ghent, Belgium) with a 30 min gradient 10–60% B followed by 3 min to 90% B (solvent system A: 100% water versus B: 100% acetonitrile, both containing 0.1% formic acid) at a flow rate of 0.3 µl/min. AKH candidate peaks were identified by target-MS/MS in positive ion mode using CID for eligible known peptides according to references^2,8^. Both the singly- and doubly-charged parent ions were measured (0.2 s scan rate). Collision energies in MS/MS experiments were run as manually optimized ramps to meet the individual fragmentation behavior (~ 12–27 eV for [M + H]^+^, slightly lower for [M + 2H]^2+^]). Screening of new extracts was performed with low/high collision energy switching (4/30 eV) in data-independent (so-called MSe) experiments in the range *m/z* 400–1500. High-energy data were interrogated for the gas phase loss of the Trp immonium ion. Data were analyzed manually. Spectra labelling follows the nomenclature used by the Waters MassLynx software; the fragment ion tables for the spectra are available in the Supplement for explanation.

*Validation of AKHs in S. cyclindricum:* Synthetic peptides Melme-CC and Dorpa-AKH were custom synthesized by Pepmic Co., Ltd (Suzhou, China) and prepared at 1 pmol/µl. Both the endogenous and the synthetic samples were spiked with bradykinin1-7 (Sigma, 1 pmol/µl stock solution) for control of the retention time, which was 25.4 min and thus about 6 min earlier than the AKHs. It allowed correction of the LC arrival time following heavy unrelated use of the instrumentation, if necessary. The peptides were run with identical parameters separated by blank runs.

*Database assembly:* Previously characterized and validated AKH primary sequences (referred to as “known AKHs” in this study) were obtained from lists available in references^2–4,8,10–12^ and individual references as listed with the entry in the Supplementary File, sheet “Known & predicted AKHs”. To compile a list of predicted sequences that have not yet been validated at the protein/amino acid level, the Uniprot database was searched using the term “adipokinetic hormone” (Feb 1, 2022). AKH-type of sequences were located in the translated nucleotide data by looking for a stretch of eight amino acid residues starting with Gln/Glu and ending with Trp. The dibasic cleavage site and the amidation signal in AKH-prohormones were not considered during this step. The amino acid sequences of the octapeptides plus the two residues following the Trp residue were imported into Excel for subsequent sequence comparison. Moreover, the pattern search function of the ProteinProspector web tool (The Regents of the University of California) was employed to screen the Uniprot database for 8-amino acid residue long sequences of the AKH format; this strategy automatically produced information for the amino acid residue in position 9, i.e. following Trp. Thereby, the known rules for AKH composition were observed^1^. All search results were manually curated to provide a searchable and sortable archive and are provided in the Supplementary File, sheets “Known AKHs” and “AKH in context”. Updated versions of the archive will be made available at this link: https://www.medizin.uni-muenster.de/cu-proteomics/projekte.html).

## Results and discussion

**Figure 2:**
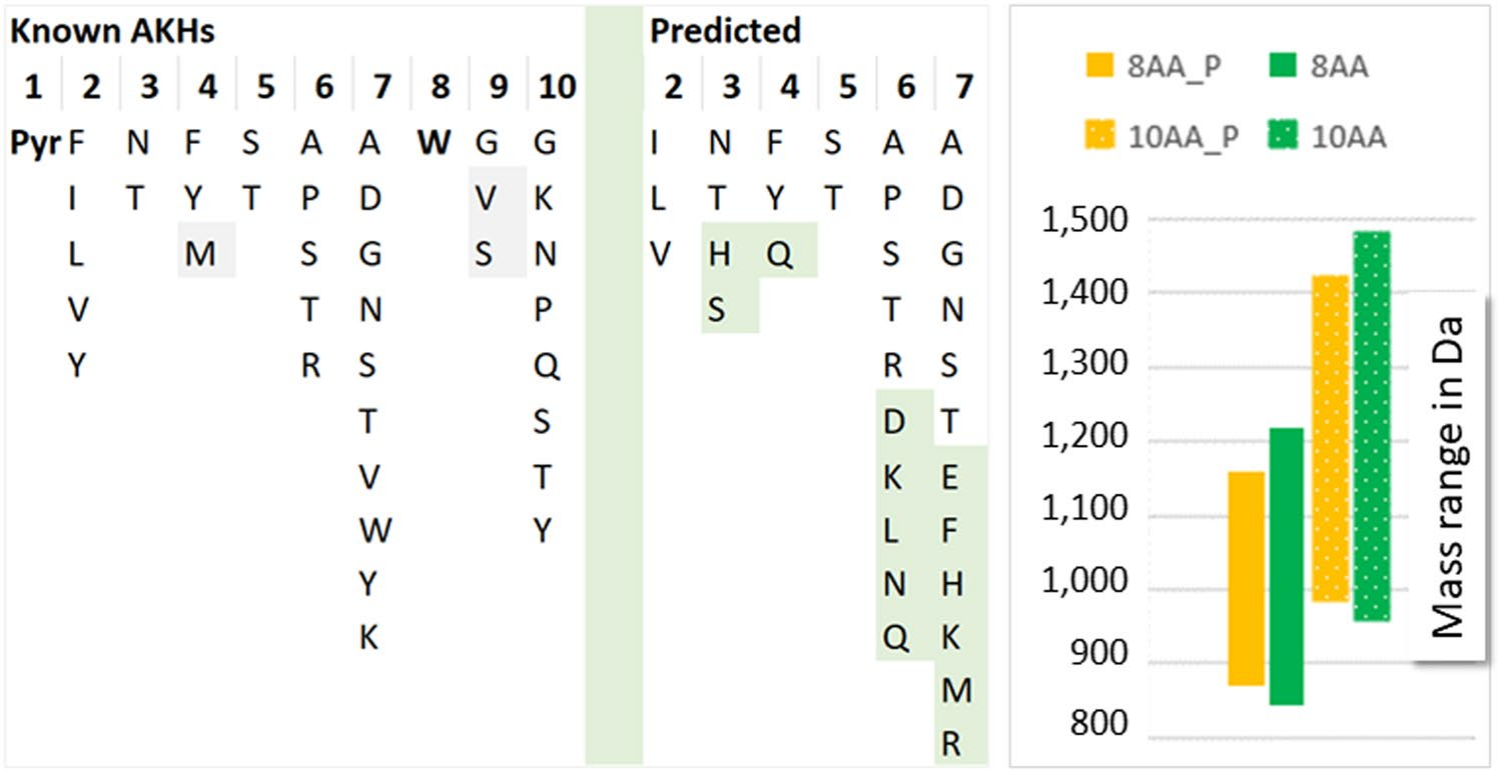
Possible amino acids in known AKHs as well as in Uniprot sequences, which contain the AKH basic sequence stretches and were obtained with the search term “adipokinetic hormone” (see Materials and Methods). Fixed positions 1 and 8 are shown in bold. Less frequently occurring amino acid residues in known AKHs are marked in grey, possible additional amino acid residues found in predicted sequences are highlighted in green. With this information the mass range (y-axis) of expected peptides was calculated for Pro6- (_P, orange) and non-Pro6 (green) peptides of 8- (8AA) and 10-amino acid (10AA) long sequences.

AKHs have conserved sequence features, which help the analyst in forming sequence hypotheses (Fig. 2). Increasingly, also genomic and transcriptomic predictions become available^5^, requiring validation for biological studies. We, thus, assembled a data resource of both known and predicted AKH sequences (see Supplement, updated versions will be made available at this link: https://www.medizin.uni-muenster.de/cu-proteomics/projekte.html) primarily meant as a resource for the analytical chemist, hence no discrimination was made with respect to species as long as the basic AKH sequence features were met. Consequently, entries from bacteria were not removed. Moreover, signal systems, namely AKHs and ACPs (AKH/corazonin-related peptides) were not differentiated. The ACPs of insects have characteristic sequences like pQxxxxRDWNA-NH_2_; for more information on this topic, see reference^1^. The downloaded data from Uniprot was manually curated and matched with the known 102 AKHs resulting in 2963 entries. It became obvious, that in the global proteome, AKH-type of sequence stretches framed by Gln/Glu and Trp are not as rare as initially suspected, especially, when more amino acids were allowed in positions 2–7 than currently assumed^1^. We allowed, for instance, also the hydrophobic Ala in position 2, since other amino acids with hydrophobic side chains (Val, Ile, Leu, Phe, Tyr) are known to occur in AKHs. Furthermore, we extended our search to include AKH-like sequences with His in position 3, for AKHs belong to the same superfamily as vertebrate GnRHs, which have His3. All search results are available in the Supplement and give an impression where in this pool of sequences the known AKHs are located.

For more specific information, we analyzed only the entries obtained with the search term “adipokinetic hormone” further. The possible amino acids in each position are summarized in Fig. 2. Search results with His in position 3 were obtained in the snails *Aplysia californica* and *Charonia tritonis*. Positions 2 and 5 seem to be truly set, while even more amino acid residues than determined so far are possible for positions 6 and 7. This data collection allows to calculate the highest and lowest expected masses of AKH peptides (Fig. 2), which may come in handy when working with unknowns to exclude unrelated mass peaks. The lower mass limit for octapeptides was 843.39 Da (pQVSQSAGW-NH_2_) and the higher 1220.55 Da (pQYHYTRWW-NH_2_); for decapeptides limits ranged between 957.44 Da (pQVSQSAGWGG-NH_2_) and 1482.68 Da (pQYHYTRWWVY-NH_2_). Peptides containing Pro in position 6 had slightly smaller mass ranges (869.40–1161.50, 983.45–1423.63 Da). We additionally provide an AKH molecular weight and ion calculator in Supplementary File, sheet “Application”.

**Figure 3:**
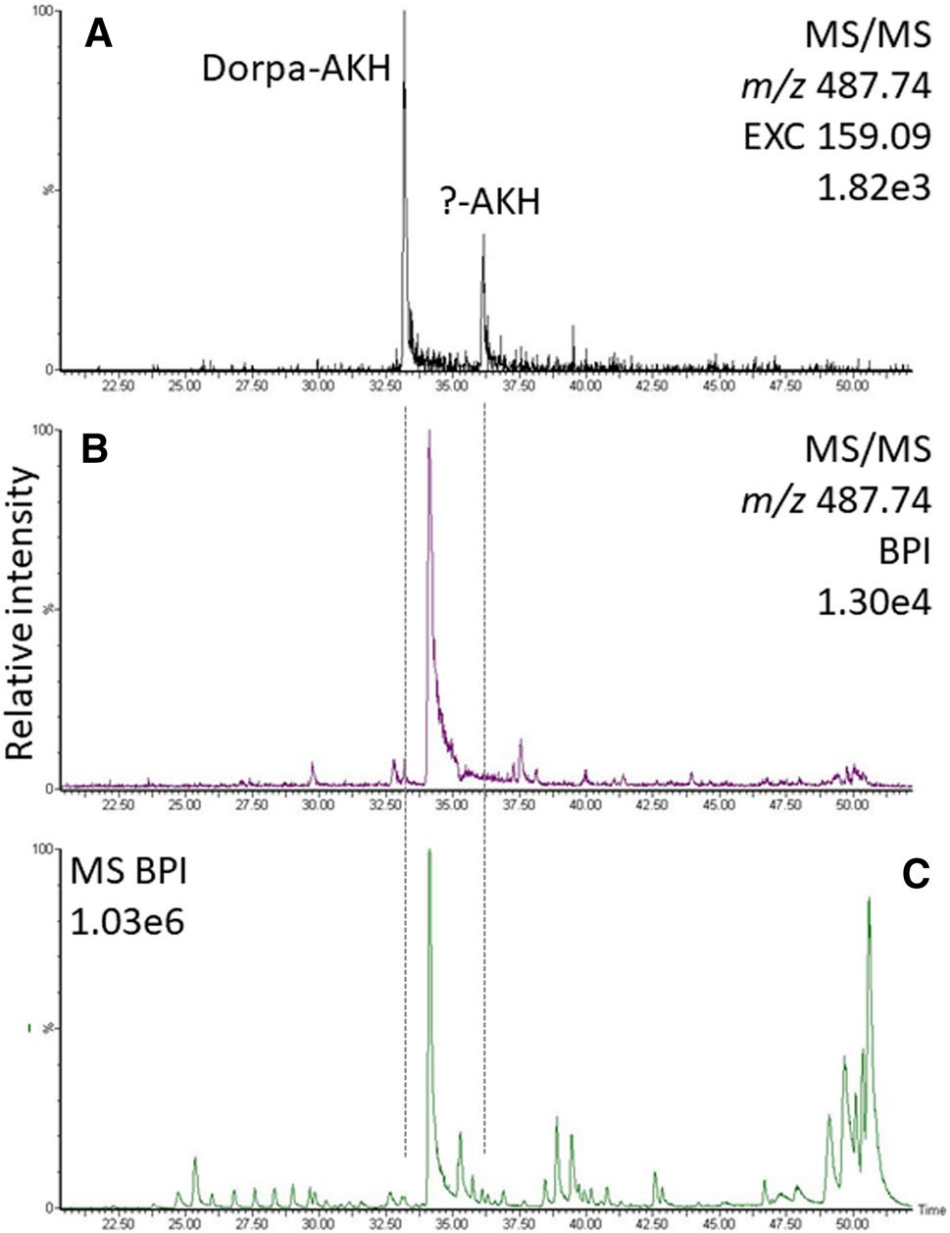
LC-chromatograms of target analyses for Dorpa-AKH ([M + H]^+^ 974.47, as measured using the doubly-charged ion at *m/z* 487.74) in the methanolic extract of CC from the scarab beetle *S. cylindricum*. (**A**) The extracted ion chromatogram (EXC) for the Trp immonium ion in the MS/MS run shows two potential AKHs. These peaks were not among the most abundant ones even in the MS/MS target trace for Dorpa-AKH (panel B; for examples of Trp immonium ion peaks, see CID spectra in Figs. 5, 6, 7, 8 and 9). The signal at 33.2 min originated from Dorpa-AKH, the second signal at 36.3 min from a potential AKH close in mass ([M + H]^+^ 973.49; for further experiments and explanations, see below). C) Base peak chromatogram showing many sample components more abundant than the AKHs. Melme-CC was also detected in this sample 0.7 min earlier than Dorpa-AKH (see below).

**Figure 4:**
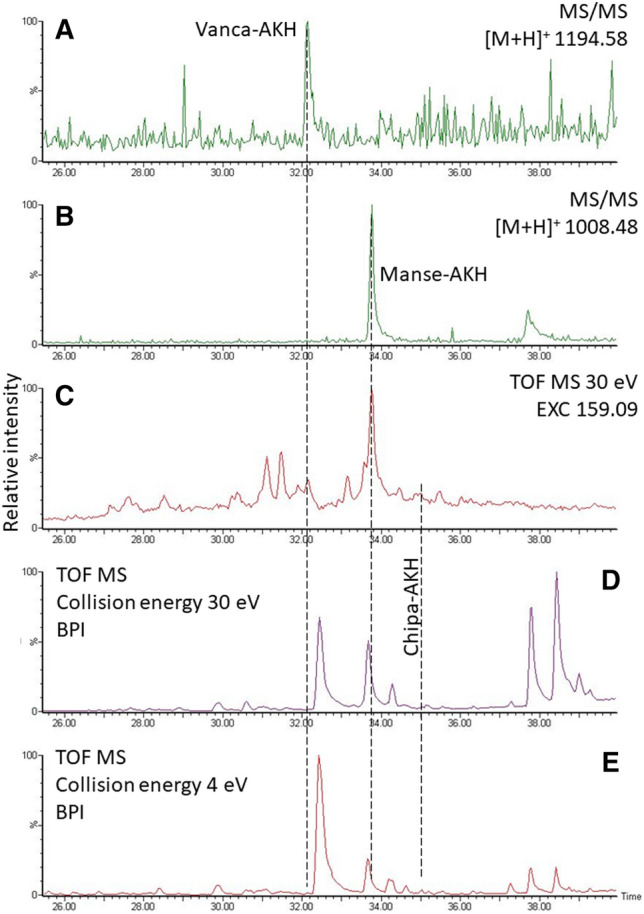
LC-chromatograms for the analysis of the CC methanolic extract of the crambid moth *C. culmella*. (**A**) Target trace for Vanca-AKH. (**B**) Target MS/MS for Manse-AKH. (**C**) EXC for the Trp immonium ion of the MS scan with 30 eV collision energy. The major peak in this trace corresponds to the peak in the target MS/MS for Manse-AKH (**B**). The two other AKHs found in this sample (Vanca-AKH, Chipa-AKH) were not immediately evident with this method. (**D**) MS scan with 30 eV collision energy. (**E**) Regular MS scan, collision energy 4 eV.

When investigating CC extracts from new species using LC–MS, the AKHs may not be immediately evident in the base peak chromatogram (for example, see, Fig. 3C) despite the fact that they are produced at significant concentrations in CCs^13^. This may be due to the sample composition, but also to factors such as ionization efficiency and matrix suppression. In the exemplary data presented in Fig. 3, the methanolic extract of the CC from the scarab beetle *S. cylindricum* was separated with RP-LC and analyzed with HRMS showing many signals, but the AKHs were not among the abundant ones, even in the target-MS/MS run for the suspected and present Dorpa-AKH (Fig. 3B). Even worse, the most abundant peaks can originate from other compounds in the sample as seen in Fig. 3 for the scarab beetle and in Fig. 4E for the crambid moth *C. culmella*.

A common feature for many AKHs in MS fragment ion spectra is the Trp immonium ion (Fig. 2). In general, Trp is the least abundant amino acid in cells and one of the rarest in the proteome^14^. It is thus worthwhile to screen for its characteristic ion using the mass trace as demonstrated in Fig. 3A for the target analyses for Dorpa-AKH ([M + H]^+^ 974.47) in the CC extract from *S. cylindricum*. The extracted ion chromatogram (EXC) for the Trp immonium ion in the MS/MS run indicated two potential AKHs. These peaks were not among the most abundant signals even in the MS/MS target trace for Dorpa-AKH (Fig. 3B). The sequences were ultimately assigned to Dorpa-AKH and a novel AKH close in mass ([M + H]^+^ 973.49, see below).

**Figure 5:**
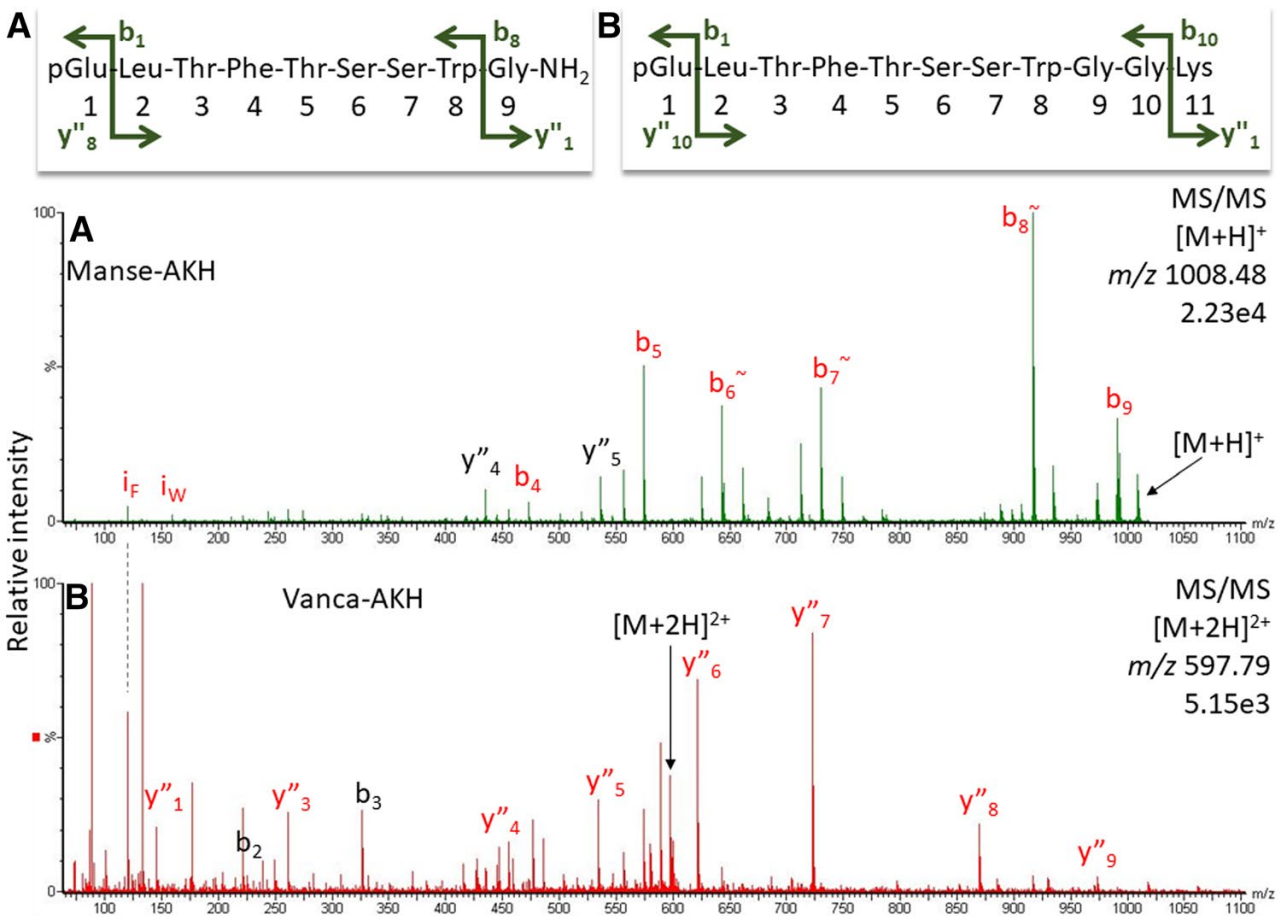
CID spectra for Manse-AKH (**A**) and Vanca-AKH (see text) (**B**) detected in the CC methanol extract of *C. culmella*. Peak labels indicate the b- and y-ion series and neutral water losses (~) as well as immonium ions for Trp and Phe (i). For explanation and fragment ion masses, see Supplement.

In data-independent measurements, screening for AKHs can also be performed by collision energy switching and subsequent search for abundant Trp immonium ions in the MS-trace of the high-energy experiment as shown in Fig. 4C/D for the CC methanolic extract of the crambid moth *C. culmella*. In the EXC, an AKH was indicated at 33.8 min, which was subsequently identified as Manse-AKH by a target MS/MS experiment^5^ (Figs. 4B, 5A). The other peaks in the EXC trace were not as prominent, but probing one at a time led to two more AKH sequences: Vanca-AKH (Figs. 4A, 5B) and Chipa-AKH (for spectrum, see^5^). Obviously, the use of the Trp immonium ion is not a one-for-all solution, but it can be useful as a next step to target screens for potentially present AKHs known from related species (here: for Lepidoptera^10^, Scarabaeoidea^2^, Diptera^8^). On a side note, the Phe immonium ion (Fig. 1) also tends to be present in Phe-containing AKHs and may thus serve the same purpose.

**Figure 6:**
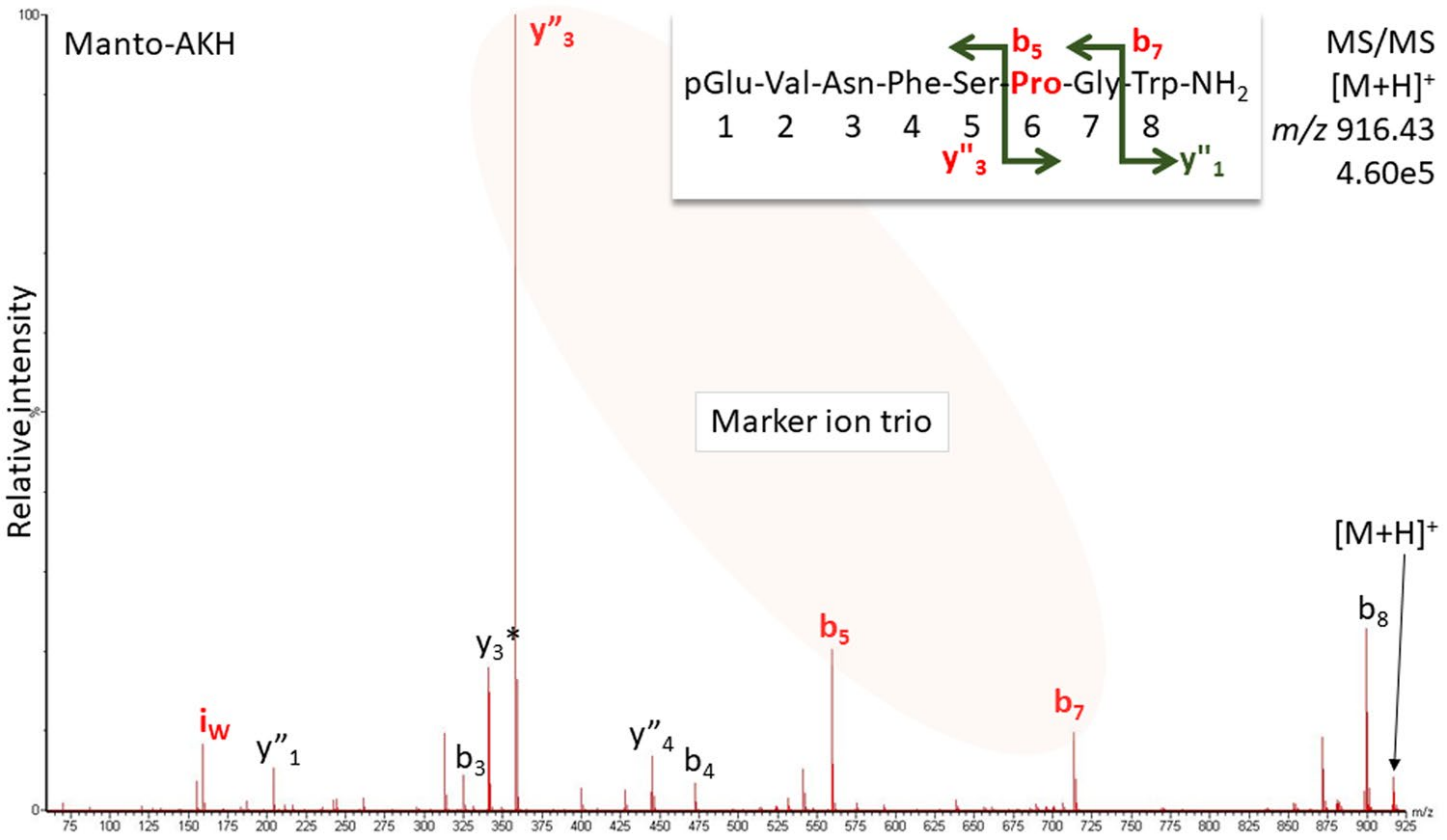
CID spectrum for Manto-CC detected in CC methanol extract of termite *K. flavicollis*. Pro in position 6 causes preferred fragmentation N-terminally to the Pro-residue resulting in a prominent y”_3_-b_5_-b_7_ ion trio in 8-amino acid residue AKHs. Peak labels indicate the b- and y-ion series and neutral ammonia losses (*) as well as the Trp immonium ion (i). For explanation and fragment ion masses, see Supplement.

**Figure 7:**
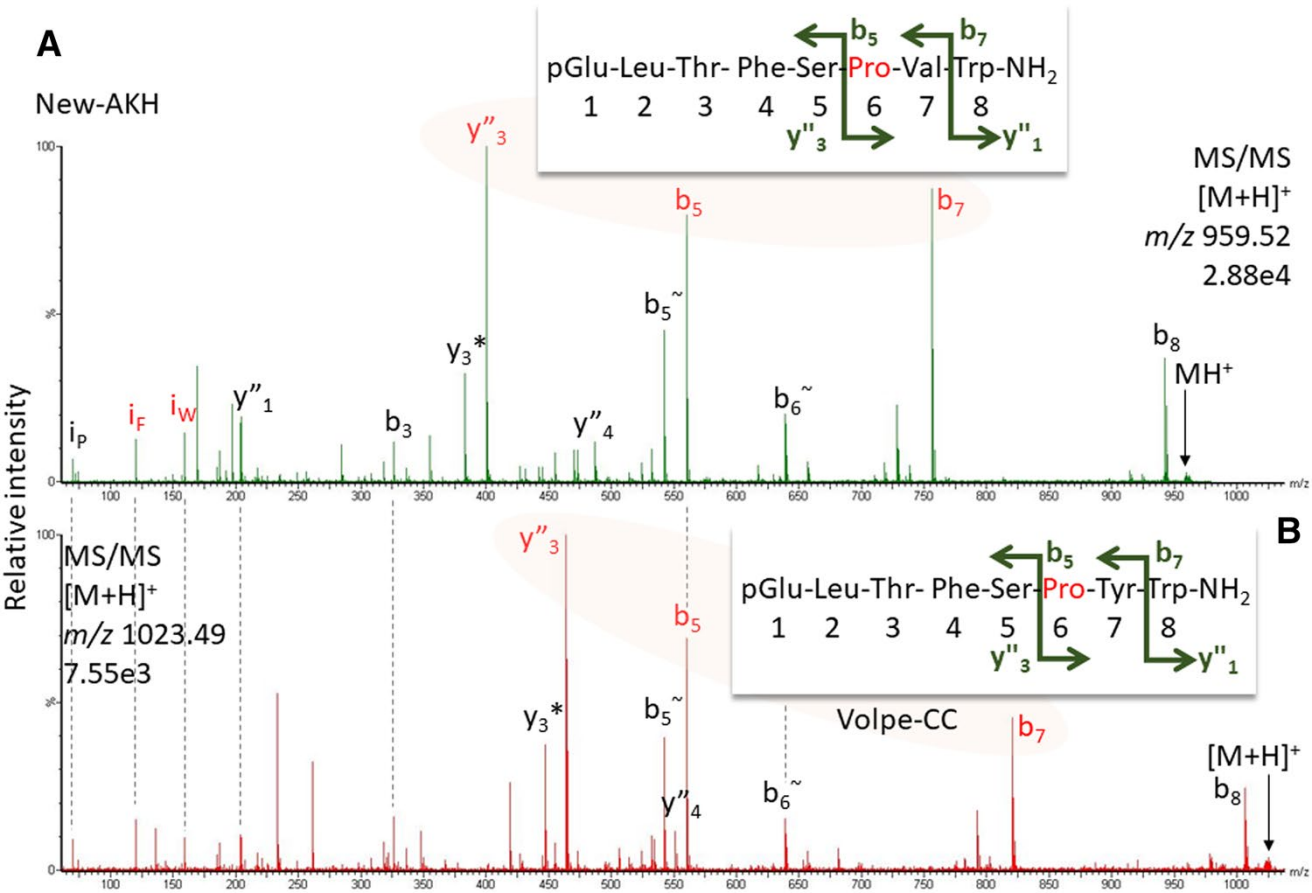
CID spectra for a new AKH code-named Pegta-AKH^5^ (**A**) similar to Volpe-CC (**B**) detected in the CC methanolic extract of the robber fly *P. tapulus*. Pro in position 6 causes a prominent y”_3_-b_5_-b_7_ ion trio. Peak labels indicate the b- and y-ion series and neutral water (~) and ammonia (*) losses as well as immonium ions for Trp and Phe (i). For explanation and fragment ion masses, see Supplement.

**Figure 8:**
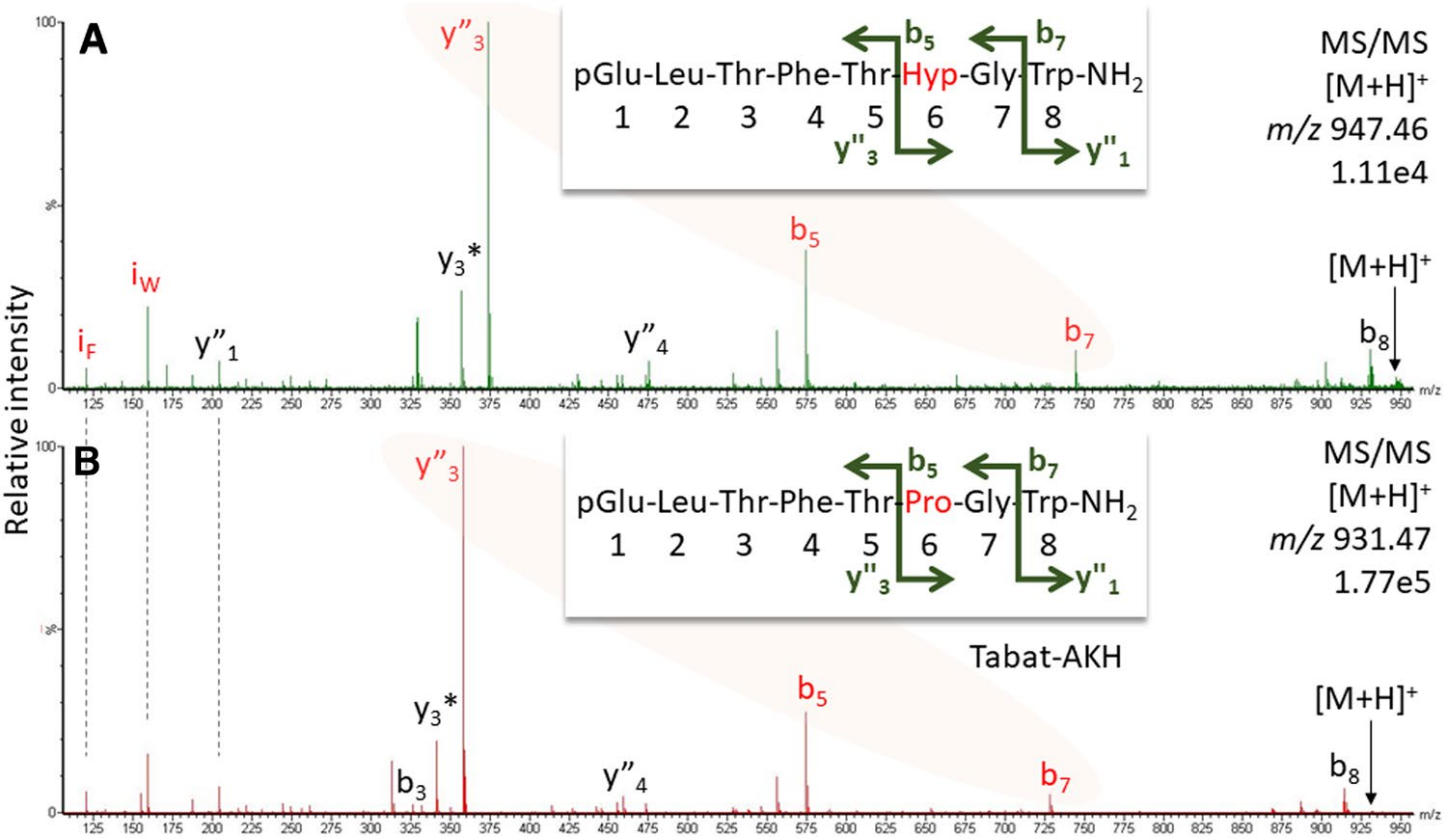
CID spectra for a new AKH code-named Haepl-AKH^5^, (**A**) and Tabat-AKH (**B**) detected in the CC methanolic extract of the horse fly *H. pluvialis*. Pro in position 6 causes a prominent y”_3_-b_5_-b_7_ ion trio, which is also true for Hyp. Peak labels indicate the b- and y-ion series and neutral ammonia losses (*) as well as immonium ions for Trp and Phe (i). For explanation and fragment ion masses, see Supplement.

**Figure 9:**
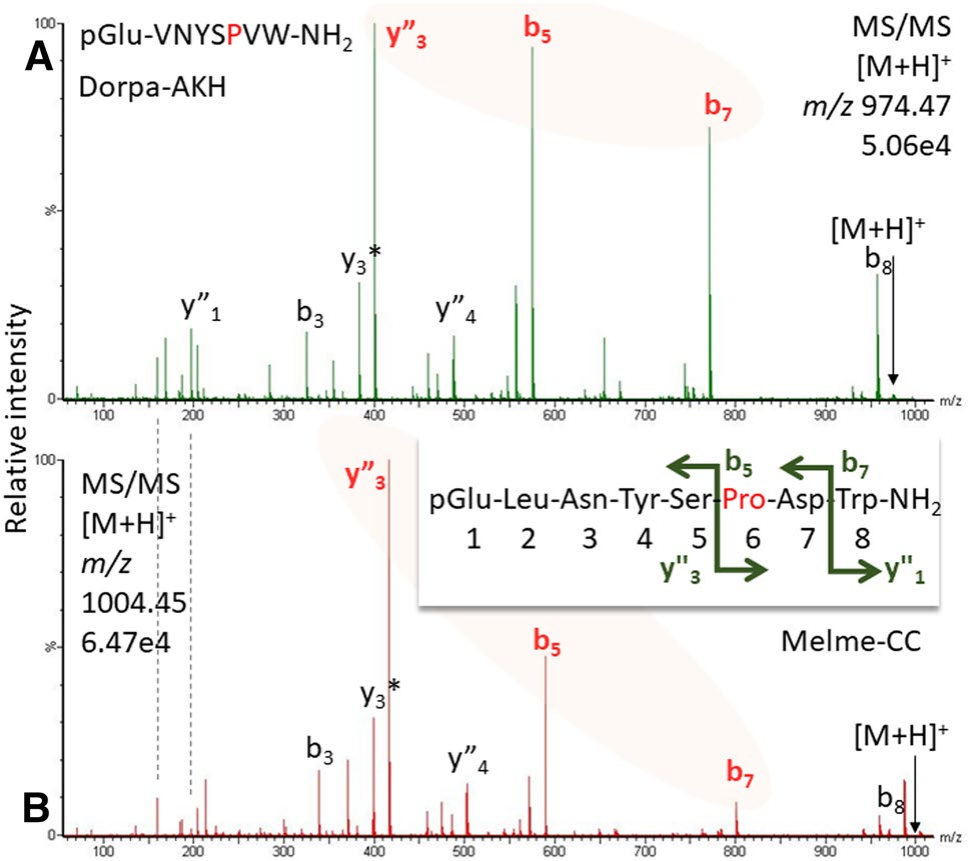
CID spectra for (**A**) Dorpa-AKH and (**B**) Melme-CC detected in the CC methanolic extract of the scarab beetle *S. cylindricum* (for validation with synthetic peptide, see Supplementary File, sheet “MSMS ions”). Pro in position 6 causes a prominent y”_3_-b_5_-b_7_ ion trio. For fragment ion masses, see Supplement.

More than half (61%) of the so far validated AKHs contain Pro in position 6. In this study alone we counted eight, which allowed us to discover the remarkable differences in fragmentation behavior between Pro6-containing AKHs and others. As is illustrated in the CID spectrum for Manto-CC (pQVNFSPGW-NH_2_, Fig. 6), which had been predicted in *K. flavicollis*^5^, the fragment ions y”_3_/b_5_/b_7_, which originated from cleavage at and around the Pro residue as sketched in Fig. 1, were dominant. This was also observed in other species for Volpe-CC (Fig. 7B), Tabat-AKH (Fig. 8B), Dorpa-AKH, and Melme-CC (Fig. 9). This ion pattern can be expected in octapeptides; for decapeptides, correspondingly, the y”_5_ ion is the prominent one. This ion is higher in mass than y”_3_ and thus close in mass to b_5_ so that the characteristic marker feature is lost as was observed for Tabat-HoTH^5^. The ion mass ranges for the three marker ions were calculated based on the current state of knowledge (search with term “adipokinetic hormones”, see Materials and Methods) and are available in the Supplement for reference (sheet “Useful charts”). Although also present in CID spectra of doubly-charged ions, the ion trio is best observed in the CID spectra of singly-charged ions due to fewer interfering peaks.

Non-Pro-AKHs, in contrast, exhibited prominent b-ions as is exemplarily illustrated with the CID spectrum for Manse-AKH (Fig. 5A). The CID spectrum for Vanca-AKH (Fig. 5B) is presented as exception; this peptide is not amidated and shows a complete y-ion series. Vanca-AKH is not considered a mature AKH, but an incompletely processed form of Manse-AKH^10^ so that its fragmentation cannot be considered as AKH-typical. Gas phase fragmentation of protonated peptides is highly dependent on their sequence; amidation in comparison to an unblocked peptide terminus does not necessarily change the fragment ion pattern much, apart from the terminal ammonia instead of water loss^15,16^.

What is a disadvantage in classical peptide analysis is beneficial for the identification of 8-amino acid residue long AKHs. For Pro-containing AKHs, the break in the fragmentation pattern was already noted in fast atom bombardment measurements ~ 40 years ago^17^ but was not exploited further. The “Pro effect”, the preference for cleavage N-terminally to Pro residues, has been extensively investigated from an energy point of view and the interested reader is directed to reference^18^ as entry into the topic. Due to the unusual ring structure of this amino acid residue, the respective y-ion was shown to be more stable than the b-oxazolone ion; enhanced proton affinity was thereby not relevant for the increased ion intensity. Recognizing the marker ion trio strengthened the assignment hypotheses for two new AKH sequences described earlier^5^. In the CC extract from the robber fly *P. tapulus*, the b_5_-ion was the same as in Volpe-CC (Fig. 7) indicating the presence of pQLTFSPxW-NH_2_. The difference to the next prominent ion, b_7_, was 196 mass units rather than the 260 for the Pro-Tyr difference seen in Volpe-CC, suggesting the presence of Val. In fact, the sequence pQLTFSPVW-NH_2_ explained the spectrum and this AKH, code-named Pegta-AKH^5^, is a variant of Glomo-AKH (pQLTFSPGW-NH_2_) known from the tsetse fly *Glossina morsitans* and the hover fly *Volucella pellucens*^8^. Likewise, based on the marker ion trio, the assignment of hydroxyproline (Hyp) in a Tabat-AKH variant (Fig. 8A, Haepl-AKH^5^), detected in the methanolic CC extract of the horse fly species *H. pluvialis* was a sensible hypothesis. This posttranslational modification had been previously validated for Panbo-RPCH (pQLNFSPGW-NH_2_) in the stink bug, *Nezara viridula*^19^.

The analysis of another potential novel AKH found in the CC extract of the scarab beetle *S. cylindricum* was more difficult. The presence of an AKH was indicated at 36.3 min in the Trp immonium extracted ion chromatogram of the target MS/MS scan for Dorpa-AKH (Fig. 3A) as a result of the coincidence that it differed only by one mass unit and was co-detected. However, the CID spectrum was not straightforward to interpret, although the –GW-NH_2_ terminus was quickly determined, and thus the likelihood of this peptide being an AKH was quite evident. It was the subsequent extensive comparative analysis of all AKH spectra of this project in order to elucidate this structure that led to the discovery of the Pro marker pattern in the first place. In contrast to Melme-CC and Dorpa-AKH, which were detected in the same sample (Fig. 9, for validation with synthetic peptides, see Supplementary File, sheet “MSMS ions”), this potential AKH did not show the marker ion trio, indicating the absence of Pro. The assignment of the spectrum for the sequence hypothesis pQ[L/I]HYSTGW-NH_2_ was very convincing and this AKH could have been a variant of Harax-AKH (pQINYSTGW-NH_2_) seen in *Harmonia axyridis*^3^. However, the validation with the synthetic peptide pQLHYSTGW-NH_2_ failed; the endogenous peptide demanded considerably more collision energy for fragmentation and its retention time in LC was 5 min earlier compared to the synthetic His3 peptide. Other sequence hypotheses are currently being tested.

In summary, for the analysis of CC extracts of new species, we found the following workflow sensible and practical:

- If peptide standards are available for the AKHs under study, it pays to determine the approximate retention time of AKHs for the given LC setup. We detected all AKHs described in reference^5^ and in this report in a 30–37 min window.
- A literature and database check for known AKH sequences in related species is useful, because chances are high that at least one identical or very similar AKH is found in the analyte extract by target MS/MS experiments; in our case it was up to three per species (ref.^5^ and this work).
- In order to find more AKHs, collision energy switching in data-independent MS experiments may bring out candidate peptides, which show prominent Trp immonium ions as was the case for Manse-AKH of the crambid moth *C. culmella.*
- AKHs tend to produce abundant singly-charged ions at ~ *m/z* 1000 in the MS scan, which sets them apart from other peptides, so that a manual search for eligible ions in MS spectra is the next step. We provide the respective mass range windows in the Supplement. We have been more successful with this method than with data-dependent MS/MS, although care should be taken to avoid signals from smaller versions of the major AKHs present as a result of in-source fragmentation. This procedure led to the elucidation of novel Pegta-AKH and a Hyp-containing AKH (Haepl-AKH) in fly samples^5^.
- In order to generate a CID spectrum of good quality for de novo sequencing, target MS/MS should be performed on both the singly- and the doubly-charged peptide ions, because sometimes only one of them produces a suitable spectrum. The collision energy needs to be properly adjusted to have ions available over the entire mass range; otherwise sequence analysis will be limited.
- In the CID spectrum, the presence of a terminal Trp residue should be established by subtracting 17.02 mass units (for the amidated C-terminus) followed by 186.08 mass units from the parent ion. In octapeptides, both of the related ions (b_7_, b_8_) tend to be present, although they may not be among the most abundant ions. In the presence of hydroxyamino acids, additional water loss may cause more intense b^~^ ions. It was this test, which suggested a novel AKH in the beetle, because b_6_^~^, b_7_^~^ and b_8_ clearly indicated the –GW-NH_2_ terminus.
- A look at immonium ion region may suggest further amino acid residues; next to the Trp immonium ion, that for Phe is also reliable.
- The spectrum can now be interrogated for the presence of the Pro marker ion trio.
- MAPSP software used in conjunction with the knowledgebase of known sequences can help to formulate sequence hypotheses. Of course, this method ignores so far unknown information prompting us to assemble an AKH database for future reference.
- The presence of isomeric amino acids (Leu, Ile) or amino acids close in mass (Lys, Gln) needs to be clarified either in dedicated experiments or by comparison to the synthetic peptides.

## Conclusion

Although AKH sequences follow certain rules, the identification of a new sequence based on the CID spectrum can be a challenge. In the course of a recent project of analyzing AKHs of six insect species (ref.^5^ and present work), we felt the need to improve our resources and prepared an Excel-based searchable database of known and potential AKH sequences. This archive is very likely not comprehensive, but contains the majority of these peptides known to date. It is extendable and its updates are available to interested parties online via this link: https://www.medizin.uni-muenster.de/cu-proteomics/projekte.html. We use this database in conjunction with mass-dependent computational sequence calculation (MAPSP^7^) for hypothesis generation.

We also noted a characteristic fragment ion trio for 8-amino acid residue Pro-containing AKHs in Q-TOF MS, which can assist in de novo sequencing. This feature is present also in spectra of longer AKHs, but is not as clearly recognizable. In addition, we found the immonium ions useful, in particular those of Trp and Phe, which are quite reliably detected in CID spectra of AKHs. With this work we provide insights and tools for the analytical chemist working in the area of AKHs. The examples shown here will facilitate future spectra assignments. In this work, we also present evidence of, and validation for, the presence of Melme-CC and Dorpa-AKH in the CC of the scarab beetle *S. cylindricum*.

Any novel peptide sequence suggested by MS needs to be validated both for the correctness of its sequence and its biological activity. Nonetheless, MS drives the field, not the least with the confirmation of predicted AKHs.

### Supplementary Information

Supplementary Information.

## Data Availability

Updated versions of the AKH database are available at this link: https://www.medizin.uni-muenster.de/cu-proteomics/projekte.html. Other data are available upon request from the corresponding author (koenigs@uni-muenster.de).

## Abbreviations

AKH: Adipokinetic hormones
CC: Corpora cardiaca
MS: Mass spectrometry
MS/MS: Tandem MS
RP: Reversed-phase
LC: Liquid chromatography
HR: High-resolution
CID: Collision-induced dissociation
EXC: Extracted ion chromatogram

## References

[1] Marco, H., Gäde, G. Adipokinetic hormone: A hormone for all seasons? *Advances in Invertebrate. (Neuro) Endocrinology* Vol. 2, Apple Acad. Press, Burlington ON, Canada, Ch. 3, 129–175 (2020).

[2] Gäde G, Šimek P, Marco HG (2016). Novel members of the adipokinetic hormone family in beetles of the superfamily Scarabaeoidea. Amino Acids.

[3] Veenstra JA (2019). Coleoptera genome and transcriptome sequences reveal numerous differences in neuropeptide signaling between species. PeerJ.

[4] König S, Albers C, Gäde G (2005). Mass spectral signature for insect adipokinetic hormones. Rapid Commun. Mass Spectrom..

[5] Marco HG, König S, Gäde G (2022). Mass spectrometric proof of predicted peptides: Novel adipokinetic peptides in insects. Molecules.

[6] Chen X, Turecek F (2005). Simple b ions have cyclic oxazolone structures. A neutralization-reionization mass spectrometric and computational study of oxazolone radicals. J. Am. Soc. Mass Spectrom..

[7] Eisenacher M, deBraaf J, König S (2006). Mass analysis peptide sequence prediction (MAPSP). Bioinformatics.

[8] Gäde G, Šimek P, Marco HG (2020). The adipokinetic peptides in Diptera: Structure, function, and evolutionary trends. Front. Endocrin..

[9] Yeoh JGC, Pandit AA, Zandawala M, Nässel DR, Davies SA, Dow JAT (2017). DINeR: Database for insect neuropeptide research. Insect Biochem. Mol. Biol..

[10] Marco HG, Šimek P, Gäde G (2020). Unique members of the adipokinetic hormone family in butterflies and moths (Insecta, Lepidoptera). Front. Physiol..

[11] Gäde G (2009). Peptides of the adipokinetic hormone / red pigment-concentrating hormone family. A new take on biodiversity. Ann. N. Y. Acad. Sci. Trends Comp. Endocrin. Neurobiol..

[12] Gäde G, Marco HG (2022). The adipokinetic peptides of Hemiptera: Structure, function, and evolutionary trends. Front. Insect Sci..

[13] Spring JH, Gäde G (1991). Storage and release of neuropeptides from the corpus cardiacum of the Eastern lubber grasshopper, *Romalea microptera*. J. Exp. Zool..

[14] Barik S (2020). The uniqueness of tryptophan in biology: Properties, metabolism, interactions and localization in proteins. Int. J. Mol. Sci..

[15] Mouls L, Subra G, Aubagnac J-L, Martinez J, Enjalbal C (2006). Tandem mass spectrometry of amidated peptides. J. Mass Spectrom..

[16] Paizs B, Suhai S (2004). Fragmentation pathways of protonated peptides. J. Mass Spectrom..

[17] Witten JL, Schaffer MH, O’Shea M, Cook JC, Hemling ME, Rinehart KL (1984). Structures of two cockroach neuropeptides assigned by fast atom bombardment. Biochem. Biophys. Res. Comm..

[18] Raulfs MDM, Breci L, Bernier M, Hamdy O, Janiga A, Wysocki V, Poutsma JC (2014). Investigations of the mechanism of the “proline effect” in tandem mass spectrometry experiments: The “pipecolic acid effect”. J. Am. Soc. Mass Spectrom..

[19] Gäde G, Šimek P, Marco HG (2011). An invertebrate [hydroxyproline]-modified neuropeptide: Further evidence for a close evolutionary relationship between insect adipokinetic hormone and mammalian gonadotropin hormone family. Biochem. Biophys. Res. Comm..

