# Publisher Correction: The proline effect and the tryptophan immonium ion assist in de novo sequencing of adipokinetic hormones

**DOI:** 10.1038/s41598-023-42799-3

**Published:** 2023-09-18

**Authors:** Simone König, Heather G. Marco, Gerd Gäde

**Affiliations:** 1 IZKF Core Unit Proteomics, University of Münster, 48149 Münster, Germany; 2 Department of Biological Sciences, University of Cape Town, ZA‑7701 Rondebosch, Cape Town, South Africa

Correction to: *Scientific Reports* 10.1038/s41598-023-38056-2, published online 05 July 2023

In the original version of this Article, Heather G. Marco was incorrectly affiliated with ‘IZKF Core Unit Proteomics, University of Münster, 48149, Münster, Germany’. The correct affiliation is:

Department of Biological Sciences, University of Cape Town, ZA-7701 Rondebosch, Cape Town, South Africa

The original Article has been corrected.

